# Newly developed quantitative transactivation system shows difference in activation by *Vitis* CBF transcription factors on DRE/CRT elements

**DOI:** 10.1186/1746-4811-10-32

**Published:** 2014-10-03

**Authors:** Annette Nassuth, Mahbuba Siddiqua, Huogen Xiao, Michelle A Moody, Chevonne E Carlow

**Affiliations:** Department of Molecular and Cellular Biology, University of Guelph, Guelph, Ontario N1G 2 W1 Canada

**Keywords:** CBF, DRE/CRT, Agroinfiltration normalization, Transactivation effector and reporter plasmids

## Abstract

**Background:**

Agroinfiltration-based transactivation systems can determine if a protein functions as a transcription factor, and via which promoter element. However, this activation is not always a yes or no proposition. Normalization for variation in plasmid delivery into plant cells, sample collection and protein extraction is desired to allow for a quantitative comparison between transcription factors or promoter elements.

**Results:**

We developed new effector and reporter plasmids which carry additional reporter genes, as well as a procedure to assay all three reporter enzymes from a single extract. The applicability of these plasmids was demonstrated with the analysis of CBF transcription factors and their target promoter sequence, DRE/CRT. Changes in the core DRE/CRT sequence abolished activation by *Vitis* CBF1 or *Vitis* CBF4, whereas changes in the surrounding sequence lowered activation by *Vitis* CBF1 but much less so for *Vitis* CBF4. The system also detected a reduction in activation due to one amino acid change in *Vitis* CBF1.

**Conclusions:**

The newly developed effector and reporter plasmids improve the ability to quantitatively compare the activation on two different promoter elements by the same transcription factor, or between two different transcription factors on the same promoter element. The quantitative difference in activation by VrCBF1 and VrCBF4 on various DRE/CRT elements support the hypothesis that these transcription factors have unique roles in the cold acclimation process.

**Electronic supplementary material:**

The online version of this article (doi:10.1186/1746-4811-10-32) contains supplementary material, which is available to authorized users.

## Background

Transient transactivation systems have been developed to evaluate the activation of different promoters by transcription factors. They have been used successfully to analyze relative promoter strengths [1, 2], which are reportedly similar to those in transgenic systems [3]. Transient expression systems are preferred for the analysis of the sequence targeted by a transcription factor because genes that are directly activated by the transcription factor will produce transcripts within the time period between infiltration and harvest, whereas genes that are indirectly activated will take a longer time. Synthetic promoters containing (multiple copies of) defined regulatory elements are often used to avoid complications due to a combinatorial effect of various elements in a natural promoter, and have allowed the confirmation of cis-regulatory elements important for promoter activation by pathogens [4, 5].

DNA from promoter- and transcription factor-plasmids of interest have been introduced into plant cells by a wide variety of means but the most successful methods involve electroporation or PEG treatment for introduction into protoplasts [6–8], and particle bombardment [9, 10] or agroinfiltration for introduction into plant tissues [11, 12]. Studies on grape genes have employed particle bombardment of grapevine callus to investigate transactivation by transcription factors [13] and, with varying success, vacuum infiltration of grapevine leaves from *in vitro* grown plants to investigate gene function in fungal defense [14, 15] or agroinfiltration to study subcellular localization [16] or silencing constructs [17].

The CBF pathway in plants ultimately results in the expression of cold regulated (*COR*) genes which encode proteins that are thought to help the plant survive frost [18, 19]. The name of the pathway derives from CBFs (CRT binding factors), the transcription factors initially discovered in Arabidopsis to be directly responsible for the activation of many *COR* genes at low temperatures by binding to CRT (defined as GCCGAC) elements in their promoters [20, 21]. The same proteins were also discovered as DRE-binding transcription factors 1 (DREB1s), reported to bind to drought responsive elements (DRE; defined as TACCGACAT) [22, 23]. As a result reference is often made to CBF/DREB1 factors (AtCBF1/DREB1B, AtCBF2/DREB1A, AtCBF3/DREB1C) that bind to CRT/DRE elements with the sequence A/GCCGAC [18]. CBF/DREB1 proteins have now been reported for a wide variety of plants [19, 24] and these appear to also bind and activate via the CRT/DRE sequence. However, not all CBF proteins have the same affinity and specificity for a certain CRT sequence. For example, the *Brassica napus* BNCBF17 has a lower sequence binding specificity than BNCBF5 [25] whereas the barley HvCBF1 has a binding preference for an element, TTGCCGACAT, containing the GCCGAC (CRT) core sequence over a sequence with the ACCGAC (DRE) core [26]. The results with Chrysanthemum *DREB1A*- or *DREB1B*-overexpressing *Arabidopsis* showed that these CBFs activate different, overlapping regulons, which is in agreement with preferences of these CBF-like proteins for different promoter elements [27]. Also analysis of the promoters from genes that were induced in AtCBF-overexpressing Arabidopsis revealed that variations in the sequence surrounding the CRT element might affect activation by various CBFs [28, 29]. Together these results suggest that different CBF paralogs in a plant, and possibly orthologs from different species, have unique preferences for CRT-like sequences but more research is needed to investigate this further. Our lab successfully applied agroinfiltration of tobacco leaves to show that CRT promoter elements are required for regulation of gene expression by grape CBF transcription factors [30, 31]. The results also suggested that CBF4 activates better than CBF1 however our analyses did not consider differences in infiltration and extraction that might occur between separate events.

The goal of the present study was to introduce an optimized dual luciferase reporter assay system that allows a better quantitative comparison of gene expression between different combinations of transcription factors (TFs) and promoter elements. The resulting system was used to analyze the activation by grape CBF1 and CBF4 on artificial promoters containing variations of the CRT sequence, and to compare the activation by CBF1 of the wild grape *Vitis riparia* (VrCBF1) and a VrCBF1 with one amino acid mutated into the amino acid present in the CBF1 of the more freezing sensitive winegrape *V. vinifera*.

## Results

### Development of a quantitative dual luciferase transactivation system

**Figure 1:**
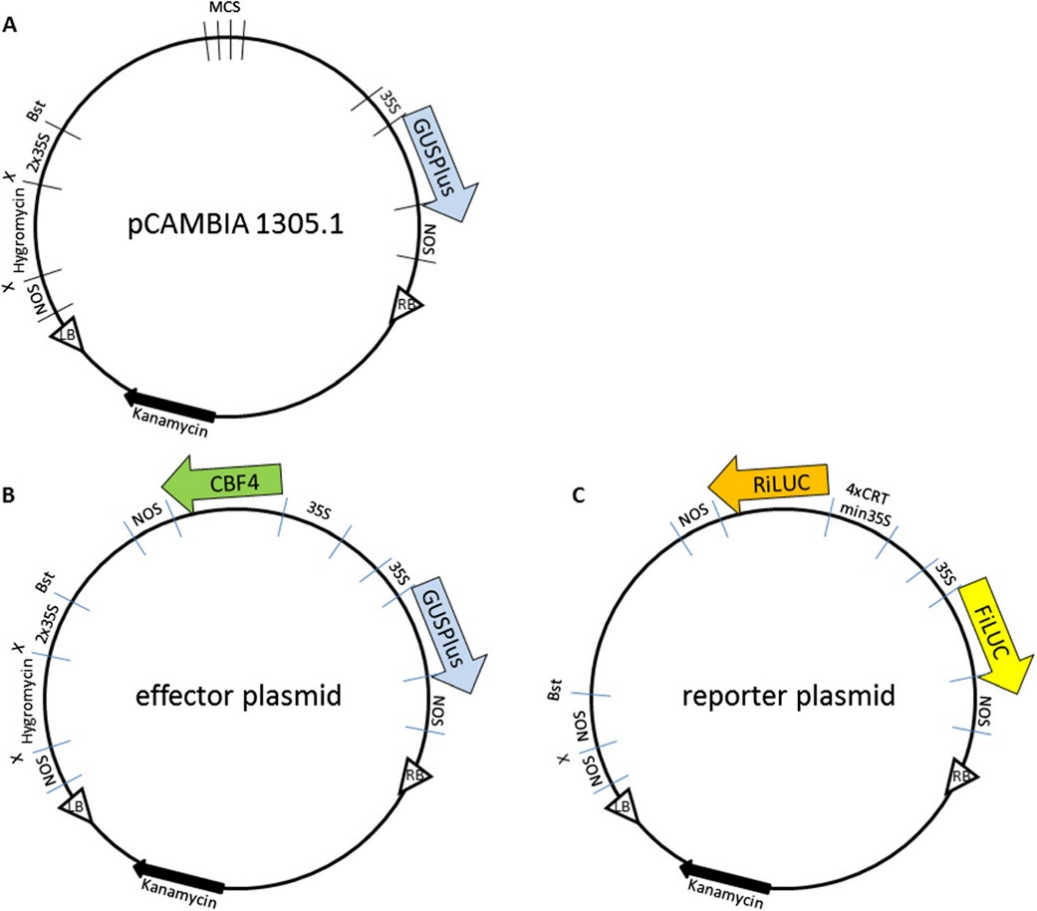
**Schematic representation of [A] pCAMBIA1305.1, [B] GUS effector and [C] dual luciferase reporter plasmids.** MCS = multiple cloning site. See Materials and Methods text for further details.

Effector plasmids were prepared starting from pCAMBIA 1305.1 (Figure 1A). For the VrCBF4 effector plasmid (Figure 1B), this involved adding a 35S::VrCBF4-nos terminator cassette into the multiple cloning site (MCS). The VrCBF4 open reading frame (ORF) was replaced by the VrCBF1 ORF for the VrCBF1 effector plasmid. To prepare the reporter plasmid, the VrCBF4 effector plasmid was altered in several aspects. The GUSPlus ORF was replaced by the firefly luciferase (FiLUC) ORF, the VrCBF4 ORF was replaced by the renilla luciferase (RiLUC) ORF, and its 35S promoter was replaced by a 4XCRTmin35S promoter (Figure 1C). As a result, the GUS activity can be taken as an indicator of the amount of effector plasmid, and the FiLUC activity as an indicator of the amount of reporter plasmid.

**Figure 2:**
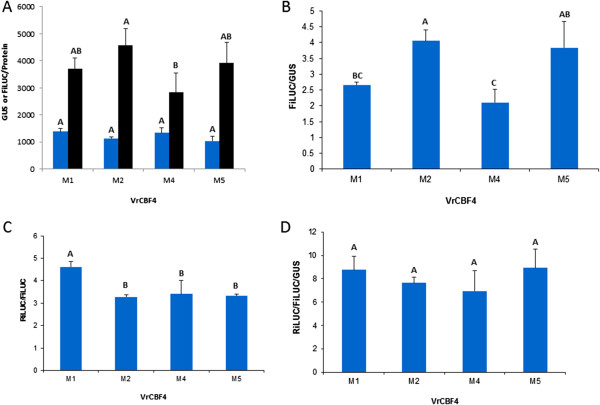
**Analysis of reporter gene expression after agroinfiltration of tobacco leaves with VrCBF4 effector in combination with four different 4XCRTmin35S reporter constructs. [A]** GUS/protein (blue bars) and FiLUC/protein (black bars), **[B]** FiLUC/GUS, **[C]** RiLUC/FiLUC, **[D]** RiLUC/FiLUC/GUS. Shown are the averages of three technical replicates and their standard deviation. Different letters indicate statistically significant differences (ANOVA p < 0.05).

Various assay conditions were tested for compatibility with an analysis of the activities of the beta-glucuronidase, renilla luciferase and firefly luciferase reporter enzymes in a single extract. It was determined that extracts prepared in CCLR and diluted in PLB buffer (both from Promega) could be used for either glucuronidase or luciferase assays. Dilutions between 75 and 100x gave values below the maximum value for the fluorescence reader and were in a linear range. This indicates that all reporter enzyme activity values could be determined from the same extract.The GUS/protein and FiLUC/protein values were expected to be the same for each effector/reporter infiltration treatment if the amount of DNA taken up and expressed in leaves infiltrated with VrCBF4 effector and reporter plasmid-containing agrobacteria (reporters containing the M1, M2, M4 and M5 variants of the DRE/CRT element, for details on these variants see a later section), was similar. The results show that both GUS/protein and FiLUC/protein values vary (Figure 2A), indicating that the amount of DNA transferred and expressed in the plant cells after the different infiltrations varied for both effector (GUS) and reporter (FiLUC) plasmid. Also the FiLUC/GUS values vary (Figure 2B), indicating that the ratio between effector DNA and reporter DNA uptake and expression varies. This means that the amounts of CBF protein and reporter gene in the cells might vary independently from each other, and this can cause variations in activation between separate infiltrations. The RiLUC/FiLUC ratio can therefore not be taken as a true measure of transactivation (Figure 2C). Instead it is better to normalize for the amount and expression of effector plasmid (represented by GUS) as well and therefore the RiLUC/FiLUC/GUS was taken as a measure of transactivation (Figure 2D).

### VrCBF1 and VrCBF4 require the conserved DRE/CRT core sequence to transactivate

**Figure 3:**
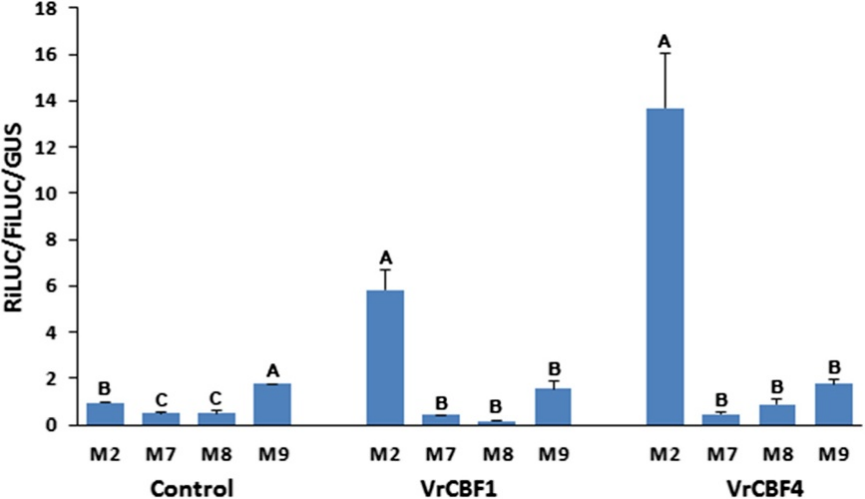
**Activation by VrCBF1 or VrCBF4 on CRT (M2) and mutated core CRT sequence.** M2: TG*CCGAC*AT, M7: TGAAGACAT, M8: TGCCGCCAT, M9: TGCCGAAAT. Error bars represent the standard deviation. Infiltrations without CBF effector were included as control. Statistical analysis was performed on the set of no CBF, VrCBF1 and VrCBF4 data separately and significantly different activation values (ANOVA p < 0.05) are indicated by different letters. Similar results were obtained for two other independent experiments.

To determine whether the transactivation by VrCBF1 and VrCBF4 has strict requirements regarding the TACCGACAT sequence present in the RiLUC reporter gene promoter, various mutations were made in this sequence and tested for transactivation. Examination of the results with the regular CRT sequence (GCCGAC = M2) showed that both VrCBF1 and VrCBF4 gave higher RiLUC/FiLUC/GUS values, respectively about 6 and 14 times higher, than the values obtained in the absence of a CBF (Figure 3). Mutations in the CCGAC core DRE/CRT sequence significantly reduced the activation by VrCBF1 and VrCBF4 to values that were not statistically different from those obtained without CBF.

**Figure 4:**
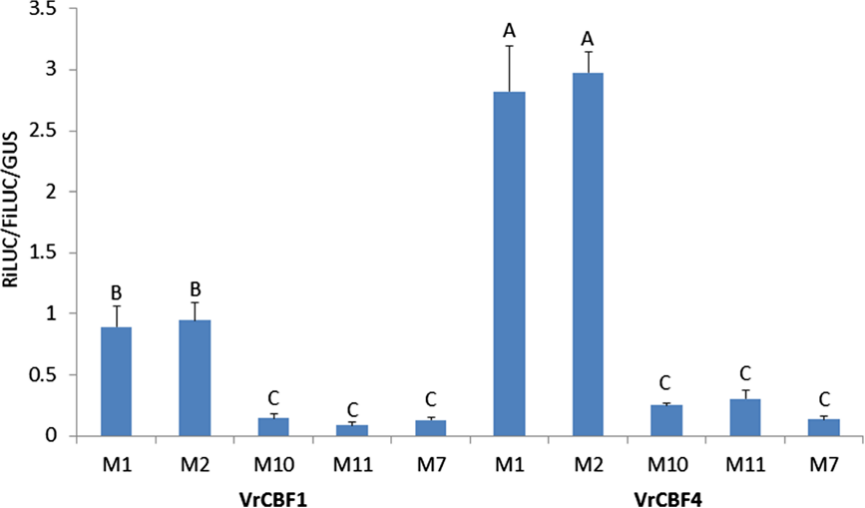
**Activation by VrCBF1 or VrCBF4 on a reporter with various initial nucleotides in the DRE/CRT sequence.** M1 = DRE: TA*CCGAC*AT, M2 = CRT: TG*CCGAC*AT, M10: TC*CCGAC*AT, M11: TT*CCGAC*AT (core CRT/DE sequence in italics), and the negative control CRT variant M7: TGAAGACAT Statistical analysis was performed on the set of no CBF, VrCBF1 and VrCBF4 data separately and significantly different activation values (ANOVA p < 0.05) are indicated by different letters. Error bars represent the standard deviation. Similar results were obtained in three other independent experiments.

**Figure 5:**
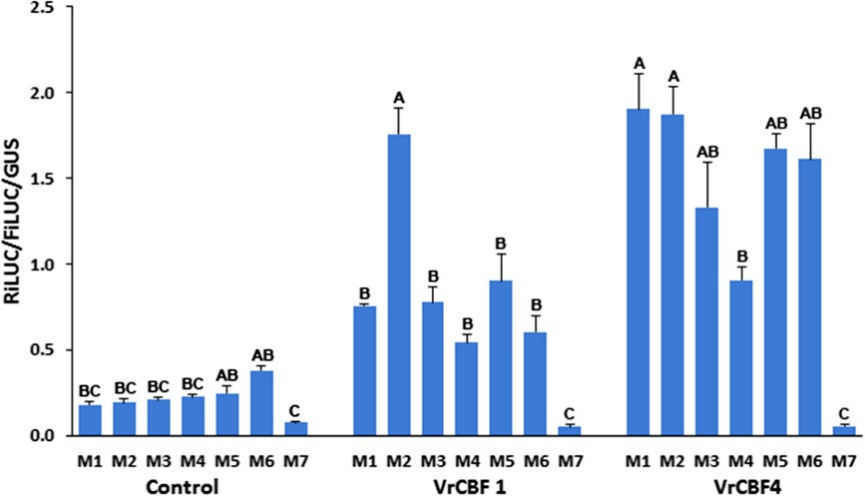
**Activation by VrCBF1 and VrCBF4 on promoters with nucleotide variations around the DRE sequence (in italics).** M1 = DRE: T*ACCGAC*AT, M2 = CRT: T*GCCGAC*AT, M3: G*ACCGAC*AT, M4: T*ACCGAC*TT, M5: G*ACCGAC*AA, M6: G*ACCGAC*TC. The M7 CRT variant (TGAAGACAT) was included as a negative reporter control, whereas mixtures without a CBF were included as a negative effector control. Error bars represent the standard deviation. Statistical analysis was performed on the set of no CBF, VrCBF1 and VrCBF4 data separately and significantly different activation values (ANOVA p < 0.05) are indicated by different letters. Similar results were obtained in two other independent experiments.

The DRE sequence is ACCGAC, whereas the CRT sequence has a G instead of an A to give GCCGAC [20–23]. The question that was posed is whether the different *Vitis* CBFs have different affinities for these 2 sequences, and whether another change of the initial nucleotide has an effect. Figure 4 shows that VrCBF1 and VrCBF4 activated reporters with either TACCGACAT (M1) or TGCCGACAT (M2) elements to similar levels, but when the first nucleotide (A or G) is mutated to C (M10:CCCGAC) or T (M11:TCCGAC) the RiLUC/FiLUC/GUS values drop to control levels. This result, and also the results from Figures 3 and 5 support our previous suggestion that CBF4 activates better than CBF1 [31]. Five independent replicates of this experiment showed a higher activation by VrCBF1 on CRT (M2) over that on DRE (M1), and this difference was significant in three experiments (see also Figure 5).

### Nucleotides flanking DRE sequence also affect activation levels

Nucleotides around the DRE sequence were changed to determine if such changes affect transactivation levels. These sequence variants were made to reflect CRT/DRE elements in Arabidopsis genes that had been reported targets of DREBs. These included elements found in genes as reported by Seki and colleagues [28], namely *RD29A/COR78*, (M1: TACCGACAT), *RD17/COR47* (M3: GACCGACAT) and *RD17/COR47* (M4: TACCGACTT). Two other chosen variants were based on the frequency logo determined for cold responsive genes, as reported by Wang and colleagues [32] (M5: GACCGACAA) or drought responsive genes (M6: GACCGACTC). In the absence of CBF effector, M1 to M6 gave variable activation values which were higher than background activation values with M7, the negative control CRT variant (Figure 5). Even higher activation values were obtained with VrCBF1 or VrCBF4 on all CRT variants except for the negative control M7. Both VrCBF1 and VrCBF4 activation on sequence variant M2 was among the highest whereas activation on sequence variant M4 was the lowest in all experiments and this was significant in two out of three independent experiments (Figure 5). However, activation by VrCBF1 was more affected by nucleotide changes around the DRE/CRT sequence than activation by VrCBF4 which resulted, generally speaking, in higher induction of transcription by VrCBF4 compared to VrCBF1 irrespective of the DRE/CRT variant present in the reporter plasmid.

### Change in CBF amino acid sequence affects activation levels

**Figure 6:**
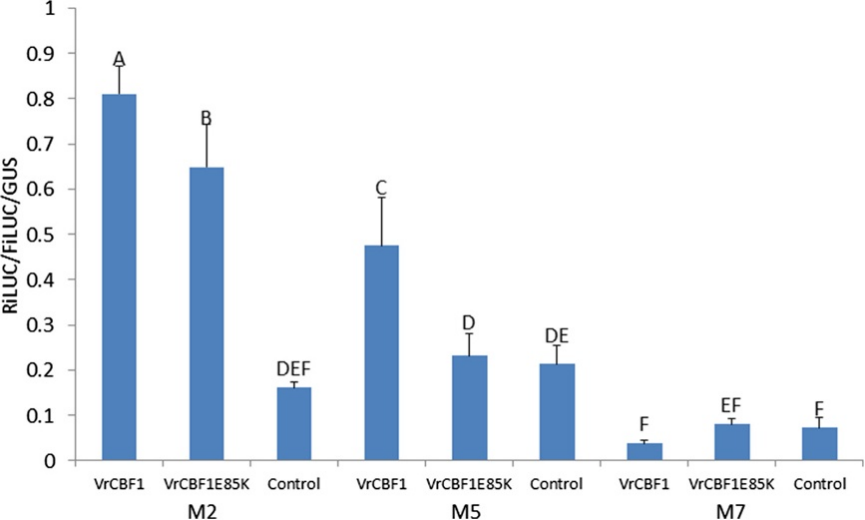
**Comparison of activation by VrCBF1and VrCBF1E85K on the M2 and M5 CRT/DRE variants.** M2 = CRT: T*GCCGAC*AT, M5: G*ACCGAC*AA. The CRT variant M7 (TGAAGACAT) was included as a negative reporter control, whereas an empty effector was included as a negative effector control. Error bars represent the standard deviation. Statistical analysis was performed on all data together and significantly different activation values (ANOVA p < 0.05) are indicated by different letters. Similar results were obtained in two other independent experiments.

Based on the higher transactivation by CBF1 from *V. riparia* compared to activation by CBF1 from *V. vinifera* in our previous transactivation system [30], we predicted that a change of the glutamic acid (E) in the AP2 DNA binding domain of VrCBF1 at position 85 to a lysine (K), as is present in VvCBF1, would decrease the transactivation. Therefore, transactivation by VrCBF1 was compared to that by the mutant VrCBF1-E85K in the newly developed dual luciferase transactivation system, on the two DRE/CRT variants that had given the highest values (M2 and M5, see Figure 5). An empty control effector plasmid was included to confirm that the activations observed with either CBF1 or CBF4 are higher than those observed in the presence of endogenous tobacco transcription factor only (control). The results showed that the mutant VrCBF1. E85K indeed had a lower activation of the reporter with either the M2 or M5 variants (4.0x or 1.1x), as compared to the wild type VrCBF1 (5.0x or 2.2x) (Figure 6). This difference in activation was observed in three independent experiments and was statistically significant in two of these.

## Discussion

New effector and reporter plasmids were developed for transactivation analyses in plant tissues. The advantages of this new transactivation system are: (1) Two reporter genes, GUSPlus and FiLUC, were included in the vector plasmids to be able to normalize for the amount of these proteins and be used as an indicator for the amount of respectively effector and reporter DNA transferred and expressed in the leaf cells. Other researchers examining either plant or animal systems have included a constitutively expressed reporter gene (35S::LUC cassette) on a separate plasmid [33, 13, 34] to normalize for differences in infiltration between samples. This was under the assumption that both plasmids are delivered in similar quantities into the cells. Our results show that this assumption is not true (Figure 2). (2) The chosen reporter genes have an intron, which cannot be spliced out by bacteria [35], therefore no enzyme is translated from erroneous transcripts that might be produced in the numerous Agrobacteria present in the infiltrated leaf tissue [36]. Commonly used reporter genes for plant tissues are β-glucuronidase (GUS) [37, 38], green fluorescent protein (GFP) [13, 39, 40], firefly (*Photinus pyralis*) luciferase (FLUC) [41], and sea pansy (*Renilla reniformis*) luciferase (RLUC) [42]. The understanding that the inclusion of an intron is important led to the development of intron-containing reporters such as GUSi [34], GUSINT [36], GUSPlus (CAMBIA, Canberra, Australia and [30]), FiLUC [43] and RiLUC [44]. (3) Beta glucuronidase (GUSPlus), renilla luciferase (RiLUC) and firefly luciferase (FiLUC) were chosen as reporters as they all can be measured by a similar procedure. We avoided green fluorescent protein (GFP) as a reporter, since this can diffuse out of the cell [9]. Previously, Renilla luciferase has been used as the normalizer [2], but studies have shown that this luciferase has a 100 fold higher signal when compared to firefly luciferase [45] which gives it a wider range. Therefore, RiLUC was chosen to quantify differences in activation for our system. (4) The quantification procedure uses the same extract for the analysis of all reporter activities. This means that the activation value, RiLUC/FiLUC/GUS, is normalized for variation that may occur through infiltration, DNA uptake or protein extraction. The fact that new substrate solution for all enzymes has to be prepared fresh for every experiment means that some variation will exist between experiments and this can affect activation values. We therefore suggest that only effector and reporter combinations that have been analyzed in the same experiment be compared to each other.

The reporter construct was designed to contain 4 DRE/CRT sequence repeats combined with a minimal 35S CaMV promoter. The 46 nt minimal 35S promoter is one of the best characterized plant core regulatory promoter domains [46, 47] with a reportedly very low basal transcription level in the absence of additional upstream regulatory elements [48], and has already been used successfully in our previous experiments [30, 31]. Although activation by transcription factors of promoters containing only 1 binding domain was shown to be detectable [49], adding additional (4) repeats of a DRE/CRT element was considered appropriate since it would give a stronger activation and therefore higher reporter enzyme activity which would make differences in activation efficiency easier to detect [4]. The low level of activation on mutant DRE/CRT reporter constructs (Figures 3 and 4) showed that there is no significant contribution from any other potential enhancer elements on the reporter vector (for example, in the 35S promoter) to the RiLUC reporter activities caused by the *Vitis* CBFs. The low RiLUC/FiLUC/GUS values on various DRE/CRT variants suggests that there is some background activation by tobacco transcription factors especially when compared to the values on the M7 mutant CRT/DRE sequence, when no grape CBF-producing plasmid was included (see especially M6 and M9, Figures 3 and 5).

An advantage of a transient transactivation system is that one can detect an increase in transcripts from genes that are directly activated by the transcription factor under study, even if this activation is temporary and therefore not detectable in transgenic plants. Another advantage is that transcripts of indirectly activated genes, which can be detected in transgenic plants, are likely absent in the transient system. The transient expression system can therefore assist to interpret results from transgenic plants. For example, we previously reported that compared to wild type Arabidopsis, VrCBF4- but not VrCBF1-overexpressing plants have increased *AtRGL3* expression [50]. One might speculate that this is due to a preference by VrCBF4 and not VrCBF1 for the DRE/CRT-like sequence CCGCC in the *AtRGL3* promoter. However, the presented transient expression results showed that the M8 reporter construct (containing CCGCC) is not activated much by either VrCBF1 or VrCBF4. Similarly, because VrCBF1- but not VrCBF4-overexpressing plants had an increased *RD29A* (*COR78*) expression, it could be argued that this was due to a preference by VrCBF1 and not VrCBF4 for the CCGAC sequence present twice in the promoter of this gene [50]. However, the transient expression system results shows that all reporter constructs containing CCGAC (M1 to M6) are activated better by VrCBF4 than by VrCBF1 (Figures 4 and 5). This suggests that the induction of *AtRGL3* or *RD29A* in the transgenic Arabidopsis is due to an indirect effect, although it is also possible that the CBFs activate these genes via a DRE/CRT element outside of the “promoter” region that was examined for sequence elements, about 1 kb upstream of the ATG start codon [50].

All presented agroinfiltrations were performed in tobacco leaves, by a relatively easy procedure. We were not successful in infiltrating leaves from grapes grown under growth chamber conditions, despite trying various methods including vacuum infiltration. Indeed, also other researchers reported their failure to do so and were only successful if in plants were grown *in vitro* [14, 15]. This has not been pursued further at this time since *in vitro* culture is labour-intensive and transactivation in grape leaves would only be necessary if one wanted to analyze the transactivation of endogenous grape genes.

Changes in the core DRE/CRT sequence greatly reduced the transactivation values (Figures 3 and 4), confirming that the complete core DRE/CRT sequence is required for binding by *Vitis* CBF1 or 4. This is in line with the report that Arabidopsis DREB1A (AtCBF3) and DREB2A bind weakly or not at all when the core CRT sequence is altered [51]. Binding by the Arabidopsis DREB proteins was not affected by changes in the surrounding sequence [51] however our transactivation results show that this is different for the *Vitis* CBFs, especially for *Vitis* CBF1 (Figure 5). Also of note, is that CRT variant M8 contains the core sequence of the GCC box (GCCGCC) which is known to interact with ERF transcription factors of the ethylene signalling pathway [52] but not with Arabidopsis DREB proteins [51] and, as shown here, also not with *Vitis* CBF1 and CBF4 (Figure 3).

Inclusion of the VrCBF4 effector plasmid generally resulted in higher activation of the DRE/CRT variants than inclusion of the VrCBF1 effector plasmid (Figures 4 and 5). This supports our suggestion that VrCBF4 is a better activator than VrCBF1 on CRT variant M1 based on previous experiments using a different transactivation system [31]. In principle, there are several possible explanations for this phenomenon besides an inherent better activation capability for the VrCBF4 protein. It is possible that different amounts of protein are produced for each because, even though we used the same 5′UTR and 3′UTR sequences for each construct, the coding sequence can also affect translation efficiency [53]. Other possible explanations include a difference in the half-life of the RNA or protein. Translation efficiency and RNA or protein stability likely differ between different tissues and conditions (e.g. ambient vs cold treatment), and the situation found here in tobacco leaves might therefore not reflect the conditions that exist when VrCBF1 and VrCBF4 are expressed in grape tissues. Quantification of CBF protein levels would be possible by Western blot analysis with antibodies specific for each CBF or to a tag added to each CBF. However, the results of the experiment shown in Figure 5 suggest that possible differences in protein quantity are not the main reason for the observed differences in activation by VrCBF1 and VrCBF4. In this experiment the same experimental parameters (bacterial cultures, tobacco plants, length of incubation etc.) were used for all reporters but not all reporters show a lower activation with VrCBF1 than with VrCBF4. A more likely explanation for these results is that VrCBF1 has a preference for the M2 sequence whereas VrCBF4 is more promiscuous. The higher activation by VrCBF1 vs. VrCBF1E85K supports the hypothesis that this amino acid difference contributes to the difference in freezing tolerance between *V. riparia* and *V. vinifera*. The ability to detect this difference shows the sensitivity of the transactivation system to detect changes in activation due to single amino acid sequence differences.

## Conclusions

Here we describe the development of a novel set of effector and reporter plasmids for transient expression studies using agroinfiltration. The use of intron-containing reporter genes allow for normalization of transactivation values for variation in plasmid entry into the plant cells, sample collection and extract preparation. The ability to distinguish between activation by plasmids with minor sequence variations in DRE/CRT promoter elements or a CBF transcription factor suggests that this system could be valuable in examining a variety of transcription factors and their putative target promoter sequences. The results with VrCBF1 and VrCBF4 activation on DRE/CRT variants suggests that these two transcription factors likely activate different overlapping sets of genes, and therefore have unique roles in cold acclimation.

## Materials and methods

### Preparation of effector and reporter plasmids

The pCAMBIA 1305.1 binary vector containing a multiple cloning site (MCS) and a 35S::GUSPlus reporter gene (http://www.cambia.org/daisy/cambia/585.html), a gene with a catalase intron, was taken as the starting point for the creation of effector constructs first (Figure 1A). This is a multicopy plasmid, in contrast to the previously used pBI121 [30, 31], and thus easier to use. The original pCAMBIA plasmid, which does not encode any CBF, was used as a negative control effector. A *Hind*III/*Eco*RI cassette containing a 35S promoter, VrCBF4 coding region with 5′ ribosome binding site (rbs), and Nos terminator sequence, obtained from a previously prepared pBI121-based effector [31], was inserted into the MCS of pCAMBIA, yielding the 35S::VrCBF4 pCAMBIA effector (Figure 1B). Preparation of 35S::VrCBF1 pCAMBIA effector plasmid involved simply replacing the BamHI/SacI fragment containing the 5′ ribosome binding site and VrCBF4 coding sequence [31] with a similar fragment containing VrCBF1 coding sequence [30].

The reporter construct was prepared from these pCAMBIA effector constructs in several steps. First the GUSPlus reporter sequence was replaced with a FiLUC (Firefly luciferase coding sequence including the PIV intron from GUS^INT^) reporter sequence. To this end, NcoI/PmlI digested pCAMBIA effector, i.e. without the GUSPlus sequence, was ligated to an NcoI/PmlI fragment containing the FiLUC sequence which had been amplified from pLUC07 [42] using primers that introduce these restriction sites (FiLUC-H-2 + NcoI: 5′AGGTAAGCCATGGAAGACGCCAA 3′ and FiLUC-C1842 + PmII: 5′TACACGTGTTACAATTTGGACTTTCCGC 3′). Second, the VrCBF4 coding region was replaced with the RiLUC reporter (*Renilla reniformis* luciferase coding sequence including a modified intron from the castor bean catalase gene). This was accomplished by ligating a BamHI/SacI RiLUC fragment amplified from RiLUC plasmid [44] using primers that introduce these restriction sites (RiLUC-H1 + BamHI: 5′ATGGATCCAAGGAGATATAACAATGACTTCGAAAGTTTATGATCC 3′ and RiLUC-C936 + SacI: 5′CGTTGACGAGCTCTTATTGTTCATTTTTGAGAACTCG 3′) to *Bam*HI and *Sac*I digested, FiLUC containing pCAMBIA, similar to the CBF fragments earlier. Third, vectors with the RiLUC reporter driven by a 4xCRTmin35S promoter, consisting of 4x TACCGACAT [30] plus 46 nucleotides of the 35S promoter [46, 47], were constructed. To this end the HindIII-BamHI 35S promoter-containing fragment was replaced with a HindIII-BamHI fragment containing 4xCRTmin35S promoter (obtained from plasmids described in [30]). Finally, the 2x35S::hygromycin fragment was deleted by digestion with *Xho*I and *Bst*XI and replaced by a nos promoter fragment amplified from pBI121 using primers that introduce these restriction sites (NosproH1+ BstXI: 5′ACCACCATGTTGGGATCATGAGCGGAGAATTAAG 3′ and NosproC307 + XhoI: 5′GCAGGCTCGAGAGATCCGGTGCAGATTATTT 3′), and the completed reporter plasmid was ready for use (Figure 1C). Effector and reporter plasmids were introduced into *A. tumefaciens* strain EHA105 according to the freeze-thaw method described by Höfgen and Willmitzer [54].

Reporter plasmids with altered CRT sequences were prepared by a quick change protocol essentially according to the procedure described by Stratagene on a smaller “35S cloning” plasmid [30] and subsequent subcloning of the new promoter fragment into the reporter plasmid. All primers that were used to prepare the various reporter plasmids are listed in Additional file 1: Table S1.

### Agroinfiltration of tobacco leaves

Agroinfiltration was performed based on the protocols developed by Bendahmane and colleagues [55] and Vaucheret [56], essentially as described previously [30, 31]. *Nicotiana benthamiana* plants were grown at 22°C for 16 hours of light and 20°C for 8 hours of dark until they reached a six leaf stage (approximately 4 weeks). Equal volumes of *A. tumefaciens* with reporter construct and of *A. tumefaciens* with effector construct were mixed to produce a final OD600 of 0.5 for each construct and the youngest two fully expanded leaves (leaves 3 and 4) of three different plants were infiltrated from the abaxial (lower) side with this mixture using a syringe. After 40-hour co-cultivation, one disc was taken from each infiltrated leaf, for a total of 6 discs per condition, frozen in liquid nitrogen and stored at -80°C. This was repeated 3–4 times to obtain 3–4 biological replicates for each mixture. Each experiment was repeated at least 2 times.

### Preparation of extracts

Several protocols were tested for the extraction of leaf tissue to identify a procedure that is compatible with both glucuronidase and luciferase activity measurements, so that all reporter enzymes could be analyzed for the same extract. The harvested, frozen leaf discs were ground to powder with liquid N_2_ and then 9 μl extraction buffer was added per mg tissue (300 μl buffer for six ~6 mm leaf discs). As extraction buffer we tried either GUS extraction buffer (25 mM potassium phosphate pH7.8, 1 mM EDTA, 7 mM 2-mercaptoethanol, 1% Triton X-100, and 10% glycerol) or 1X Cell Culture Lysis Reagent (CCLR; Luciferase assay systems, Promega). The glucuronidase and luciferase activities determined for the extracts were more consistent and higher when CCLR had been used for the preparation and PLB (Promega) for the dilution of the extracts, and therefore CCLR and PLB were used for all further experiments. Extracts were incubated on ice for 1 hour and cell debris was pelleted by centrifugation for 10 min at 13000 × g (SpeedFuge ^®^ SFR13K, Savant). The supernatant was then diluted 75x with PLB and used for protein, glucuronidase and luciferase assays.

### Analysis of protein quantities

Protein quantities were determined using the Bio-Rad dye-binding assay essentially according to the procedure described by the manufacturer (Bio-Rad), based on the method of Bradford [57], with BSA as standard.

### Analysis of GUS, RiLUC and FiLUC activities

GUS activity was determined after incubating a mixture of 10 μl of the 75x diluted sample and 90 μl of Assay buffer (50 mM pH 7 Sodium phosphate buffer, 10 mM EDTA, 0.1 Triton X-100, 0.1% N-Lauroylsarcosine Sodium Salt, 10 mM 2-mercaptoethanol, 40 mM 4-MUG) for 30 minutes at 37°C. Each reaction was stopped by adding 900 μl of 0.2 M Na_2_CO_3_ and fluorescence caused by the conversion of 4-MUG to MUG was measured in a polystyrene flatbottom 96-well plate (Sarstedt) using a POLARStar Omega (BMG Labtech) microplate reader with the excitation filter set at 360 nm and the emission filter at 460–10, and orbital shaking at 300 rpm. Each extract was analyzed in triplicate and the average measurement was taken as the value for one replicate.

The dual luciferase protocol based on the Dual Luciferase reporter assay system from Promega, as reported by Cazzonelli and colleagues [43], was used essentially unchanged to quantify the amount of RiLUC and FiLUC expression. 75x diluted sample (10 μl) was added to Luciferase reagent II (50 μl, LARII) in a polystyrene flatbottom 96-well plate (Sarstedt) and mixed by pipetting. FiLUC fluorescence, indicative of *FiLUC* gene expression, was measured immediately in a POLARStar Omega microplate reader with the setting on Luminescence (end point). To measure RiLUC fluorescence, indicative of *RiLUC* gene expression, 50 μl of Stop and Glo reagent (20 μl substrate into 1 ml S&G buffer and mixed well, freshly prepared according to the instructions from Promega) was added to each sample, mixed by pipetting and returned to the luminometer for a second measurement. Both measurements were set on 0.2 sec delay with 10 flashes per well. Activities for all replicates of each extract were measured consecutively before a second set of replicates was prepared and measured.

Transactivation was expressed as RiLUC/FiLUC/GUS x 100000. The resulting data were analyzed by a one-way analysis of variance (ANOVA). Statistical differences amongst the means for each reporter/effector combination within an experiment were determined by the Tukey–Kramer HSD tests (P < 0.05) using JMP (version 11.1.1; SAS Institute) statistical software.

## Electronic supplementary material

Additional file 1: Table S1: List of primers used to prepare effector and reporter constructs. (PDF 112 KB)
