# Supplementary material for: Newly developed quantitative transactivation system shows difference in activation by Vitis CBF transcription factors on DRE/CRT elements
Source: Plant Methods. 2014 Oct 3;10:32. doi: 10.1186/1746-4811-10-32 (PMC4196112; doi:10.1186/1746-4811-10-32)
Supplement: Supplementary file 1 — Additional file 1: Table S1: List of primers used to prepare effector and reporter constructs. (PDF 112 KB) [file 13007_2014_299_MOESM1_ESM.pdf]

Table S1. List of primers used to prepare effector and reporter constructs. Restriction site sequences are in italics

**Name of primer**	**Sequence (5′ – 3′)**	**Purpose**
FiLUC-H-2+ NcoI<br>FiLUC-C1842+PmII	AGGTAAG*CCATGG*AAGACGCCAA<br>TA*CACGTG*TTACAATTTGGACTTTCCGC	Clone FiLUC ORF into pCAMBIA reporter
RiLUC-H1+ BamHI<br>RiLUC-C936+SacI	AT*GGATCC*AAGGAGATATAACAATGACTTCGAAAGTTTATGATCC<br>CGTTGAC*GAGCTC*TTATTGTTCATTTTTGAGAACTCG	Clone RiLUC ORF into pCAMBIA reporter
NosproH1+ BstXI<br>NosproC307+ XhoI	A*CCA*CCATGT*TGG*GATCATGAGCGGAGAATTAAG<br>GCAGG*CTCGAG*AGATCCGGTGCAGATTATTT	Clone Nos promoter into pCAMBIA reporter
		Reporter starts with M1 = 4x TACCGACAT
**M2-H**<br>**M2-C**	CCGAAGCTTTGCCGACATTGCCGACATTGCCGACATTGCCGACATTGCGC*A*AGAC<br>GTCTTGCGCAATGTCGGCAATGTCGGCAATGTCGGCAATGTCGGCAAAGCTTCGG	Clone 4x TGCCGACAT into pCAMBIA reporter
**M3-H**<br>**M3-C**	CCGAAGCTTGACCGACATGACCGACATGACCGACATGACCGACATTGCGCAAGAC<br>GTCTTGCGCAATGTCGGTCATGTCGGTCATGTCGGTCATGTCGGTCAAGCTTCGG	Clone 4x GACCGACAT into pCAMBIA reporter
**M4-H**<br>**M4-C**	CCGAAGCTTTACCGACTTTACCGACTTTACCGACTTTACCGACTTTGCGCAAGAC<br>GTCTTGCGCAAAGTCGGTAAAGTCGGTAAAGTCGGTAAAGTCGGTAAAGCTTCGG	Clone 4x TACCGACTT into pCAMBIA reporter
**M5-H**<br>**M5-C**	CCGAAGCTTGACCGACAAGACCGACAAGACCGACAAGACCGACAATGCGCAAGAC<br>GTCTTGCGCATTGTCGGTCTTGTCGGTCTTGTCGGTCTTGTCGGTCAAGCTTCGG	Clone 4x GACCGACAA into pCAMBIA reporter
**M6-H**<br>**M6-C**	CCGAAGCTTGACCGACTCGACCGACTCGACCGACTCGACCGACTCTGCGCAAGAC<br>GTCTTGCGCAGAGTCGGTCGAGTCGGTCGAGTCGGTCGAGTCGGTCAAGCTTCGG	Clone 4x GACCGACTC into pCAMBIA reporter
**M7-H**<br>**M7-C**	CCGAAGCTTTGAAGACATTGAAGACATTGAAGACATTGAAGACATTGCGCAAGAC<br>GTCTTGCGCAATGTCTTCAATGTCTTCAATGTCTTCAATGTCTTCAAAGCTTCGG	Clone 4x TGAAGACAT into pCAMBIA reporter
**M8-H**<br>**M8-C**	CCGAAGCTTTGCCGCCATTGCCGCCATTGCCGCCATTGCCGCCATTGCGCAAGAC<br>GTCTTGCGCAATGGCGGCAATGGCGGCAATGGCGGCAATGGCGGCAAAGCTTCGG	Clone 4x TGCCGCCAT into pCAMBIA reporter
**M9-H**<br>**M9-C**	CCGAAGCTTTGCCGAAATTGCCGAAATTGCCGAAATTGCCGAAATTGCGCAAGAC<br>GTCTTGCGCAATTTCGGCAATTTCGGCAATTTCGGCAATTTCGGCAAAGCTTCGG	Clone 4x TGCCGAAAT into pCAMBIA reporter
**M10-H**<br>**M10-C**	CCGAAGCTTTCCCGACATTCCCGACATTCCCGACATTCCCGACATTGCGCAAGAC<br>GTCTTGCGCAATGTCGGGAATGTCGGGAATGTCGGGAATGTCGGGAAAGCTTCGG	Clone 4x TCCCGACAT into pCAMBIA reporter
**M11-H**<br>**M11-C**	CCGAAGCTTTTCCGACATTTCCGACATTTCCGACATTTCCGACATTGCGCAAGAC<br>GTCTTGCGCAATGTCGGAAATGTCGGAAATGTCGGAAATGTCGGAAAAGCTTCGG	Clone 4x TTCCGACAT into pCAMBIA reporter
